# Comparative Study of the Immune Response Induced by an Argentinian Classical Strain of IBDV in Two Chicken Breeds

**DOI:** 10.1155/2022/6255367

**Published:** 2022-11-29

**Authors:** Juan Jaton, María Soledad Lucero, Matías Richetta, Silvina Pinto, María José Gravisaco, Analía Berinstein, Evangelina Gómez, Silvina Chimeno Zoth

**Affiliations:** ^1^Laboratorio de Inmunología y Vacunas Aviares, Instituto de Agrobiotecnología y Biología Molecular, INTA-CONICET, Buenos Aires, Argentina; ^2^Gerencia de Gestión Estratégica de Procesos Complementarios, Centro de Investigación en Ciencias Veterinarias y Agronómicas, INTA, Buenos Aires, Argentina; ^3^Cátedra de Patología, Facultad de Ciencias Veterinarias, Universidad de Buenos Aires, Buenos Aires, Argentina

## Abstract

The hybrid chicken Negra INTA, which originated at the National Institute of Agricultural Technology (INTA), is the product of the cross between Barred Plymouth Rock females and Rhode Island Red males, and it is used as a laying hen for egg consumption. It has been characterized by productive parameters, but the characterization from an immunological perspective has not been done yet. Infectious bursal disease virus (IBDV) causes a highly contagious viral disease that affects the bursa of Fabricius. Although most chickens are regularly vaccinated against IBDV, this virus still generates negative impacts on production with significant economic losses. The aim of the present work was to compare the immune responses of the Negra INTA hybrid and the White Leghorn layer line to the infection with a field isolate of IBDV. Four-week-old chickens were infected with a single dose of IBDV and at 3, 5, 7, and 30 days postinfection (dpi), bursae were removed, and different parameters were evaluated. Results showed that the reduction of the bursa body (BB) ratio and the histopathological damage were maximum on day 7 postinfection (pi). The viral load was greater in the hybrid Negra INTA at 5 dpi. The humoral immune response between both breeds was similar, although more animals from the commercial line showed higher titers of neutralizing antibodies. Flow cytometry analysis revealed that Bu+ bursal lymphocytes reached a minimum at 7 dpi. Meanwhile, T cell infiltration measured by the percentage of CD3+, CD4+, and CD8+ cells in the bursa was at its maximum at 5 dpi. To our knowledge, this work describes for the first time the pathogenesis and the immune response caused by an Argentinian IBDV isolate in two different chicken lines.

## 1. Introduction

Infectious bursal disease (IBD) is an immunosuppressive, worldwide distributed disease of young chickens first reported in Gumboro, Delaware, United States, and was designated infectious bursal disease (IBD) due to morphologic and histological changes observed in the bursa of Fabricius [1]. Its etiological agent is the infectious bursal disease virus (IBDV), a nonenveloped bisegmented double-stranded RNA virus, member of the Birnaviridae family [2, 3]. Two serotypes have been recognized: serotype 1 causing disease in chickens (*Gallus gallus*) and serotype 2 which is apathogenic in chickens but causes disease in turkeys (*Meleagris gallopavo*) [4, 5]. It has been classified in various ways, but the current classification is based on genogroups [6]. IBDV is highly infectious in young animals and causes the destruction of lymphoid organs, particularly the bursa of Fabricius (BF), which is the site where B-lymphocyte maturation and differentiation occur. The target cell is the naïve B-lymphocyte, and the infection, when not fatal, causes immunosuppression [7]. IBDV is responsible for important economic losses in the poultry industry both directly, through clinical signs and mortality, and indirectly, due to failure in vaccination programs and incremented susceptibility to other pathogens [8, 9].

Several measures are usually implemented to prevent the occurrence of pathogens in flocks. Such measures include strict hygiene, effective immunization schedules, maternally derived antibody (MDA) induction, and genetic selection [10–12]. Many chicken breeds have been reported to exhibit natural resistance to various pathogens or a longer survival in intensive poultry farming conditions [13–18]. Particularly for IBDV, layer hens have been shown to be more susceptible compared to broilers (white Plymouth Rock and commercial line) in terms of clinical disease, mortality, lesions, and viral load [19, 20]. In addition, it has been demonstrated that the mortality rate, viral load, and histopathological damage caused by the infection change according to the genetic line [21–25]. Furthermore, it was shown that differences in the cellular immune response against IBDV in various breeds can be observed long after infection [26]. The discovery of genetic breeds or hybrids resistant to different pathogens present in poultry farms is of utmost importance because it offers a good prospect of reducing the expenditure on prophylactic and vaccination programs.

Backyard poultry raising provides a source of food and income for rural households in many low-income countries [27, 28]. In Argentina, the hybrid called Negra INTA, which arises from the cross between red Rhode Island males and Plymouth Rock barred females, is used for small-scale egg production. This hybrid was generated at INTA-EEA Pergamino (Instituto Nacional de Tecnología Agropecuaria, Estación Experimental Agropecuaria Pergamino), and it is distributed by the PROHUERTA program to small producers and families for the self-consumption of eggs. The productive parameters of this hybrid have been described [29]. Nevertheless, its resistance or susceptibility to IBD or any other diseases has not been studied yet. The aim of the present study was to evaluate the pathogenesis and the immune response caused by a field isolate of IBDV in the INTA hybrid and a commercial line.

## 2. Materials and Methods

### 2.1. Experimental Chickens

One-day-old White Leghorn and Negra INTA chickens were acquired from Camila farm (Suipacha, Buenos Aires, Argentina) and INTA-EEA Pergamino (Pergamino, Buenos Aires, Argentina), respectively.

### 2.2. Virus

A classical infectious bursal disease virus (cIBDV), belonging to genogroup 1, isolated from commercial establishments was used for all the experiments. The virus was kindly provided by Dr. Ariel Vagnozzi (IVIT-INTA, Buenos Aires, Argentina).

### 2.3. Viral Amplification

Seven-day-old SPF embryonated eggs (Instituto Rosenbusch S.A.) were inoculated in the yolk sac [30] with cIBDV and incubated for seven days at 37°C in an automatic incubator (Yonar, CABA, Argentina). During this time, dead embryos were stored at 4°C. Embryos were homogenized and resuspended in phosphate-buffered saline (PBS) supplemented with 100 U/ml penicillin, 100 g/ml streptomycin, and 2% glycerol. After clarification, the supernatant was filtered through 0.20 *μ*m and the viral stock was stored at −80°C until use.

### 2.4. Viral Titration

Seven-day-old SPF embryonated eggs (Instituto Rosenbusch S.A.) were inoculated with ten-fold serial dilutions of the viral stock (five eggs per dilution) and incubated at 37°C in an automatic incubator (Yonar, CABA, Argentina) for 7 days. Deaths occurring within 24 h postinoculation were considered as nonspecific and were not included in the calculation. The viral titer was expressed as the median embryo infective dose per ml (EID_50_/ml), using the Reed and Muench method [31].

### 2.5. Experimental Procedure

Forty-eight 4-week-old White Leghorn (WL) and 48 4-week-old Negra INTA (NI) chickens were randomly divided into two groups per breed. One group (*n* = 48, 24 of each breed) was inoculated with 10^3^ ELD_50_ of cIBDV by the oral route, while the other group (*n* = 48, 24 of each breed) received sterile phosphate-buffered saline (PBS) as a negative control. Control groups were kept in a separate room. Six chickens per group were sacrificed at 3, 5, and 7 days postinfection (dpi). The remaining six chickens were kept until day 28 pi for clinical observation and serological evaluation. These four groups of six chickens were again divided: four birds were intramuscularly inoculated into each leg with 200 *μ*l of a commercial live-attenuated Newcastle disease virus (NDV) vaccine (3V-Plat, Platalab S.A.) and the other two remained as negative controls for NDV.

Food (provided by Metrive S.A., Buenos Aires, Argentina) and water were provided *ad libitum*.

### 2.6. Clinical Signs, Gross Analysis, and Sample Processing

Chickens were daily monitored for any anomalies. On days 3, 5, 7, and 28 pi, postmortem examinations were carried out to evaluate pathological changes, body weight, and bursal weight. Bursae were harvested, observed for lesions, weighed, and cut into three pieces. One piece was submerged in TransZol solution (TransGen Biotech, Beijing, China) for RNA extraction. Another piece was submerged in RPMI culture medium (Gibco, Grand Island, NY, USA) for lymphocytes isolation, and the remaining piece was submerged in 10% formalin for histopathological analysis. Sera were collected at 0, 7, 14, 21, and 28 dpi for the detection of anti-IBDV and anti-NDV specific antibodies by ELISA.

### 2.7. Bursa to Body Weight Ratio

Body and bursa weights were used to calculate the bursa to body weight ratio according to the following formula: BB ratio = [bursa weight (g)/body weight (g)] × 1000 [32].

### 2.8. Histopathological Analysis

Bursal samples were placed in 10% neutral buffered formalin and paraffin embedded. Sections of BF were stained with haematoxylin and eosin following standard histological procedures and microscopically examined for the presence of bursal lesions under light microscopy. The evaluated lesions were lymphoid depletion (LD), fibrosis (F), inflammatory cell infiltration (II), edema (E), necrosis (N), intraepithelial cysts (IC), and intrafollicular cysts (IC). The severity of each lesion was determined by evaluating each lesion in 5 fields at 100x and scoring them from 1 to 5, where 1 = normal BF, 2 = <25%, 3 = 25–50%, 4 = 50–75%, and 5 = 75–100% of affected tissue.

Recovery of bursal follicles was evaluated by PAS staining [33]. Briefly, 10 fields were observed at 400x magnification, and the number of follicles with and without PAS-positive membranous structures was counted. Then, the recovery index (RI) was calculated using the following formula:

(1) $$\begin{array}{c} \text{RI}=\frac{\text{Total number of PAS}-\text{negative follicles}}{\text{Total number of PAS}-\text{positive follicles}}\text{.} \end{array}$$

### 2.9. Flow Cytometry

Lymphocytes were isolated from bursal samples and used to study T and B cells by flow cytometry, as previously described [34]. Briefly, bursae were cut in very small pieces and mechanically disrupted by pressing with a syringe plunger, in RPMI 1640. Then, cellular suspensions were passed through a 40 *μ*m·mesh (cell strainer, BD) and mononuclear cells were isolated by centrifugation over a Histopaque density gradient (1.077 g/ml; Sigma, St. Louis, MO) at room temperature. Cells were recovered from the interface and washed, and live cells were counted using trypan blue exclusion.

Cells were diluted in staining buffer, and 1 × 10^6^ cells per well were seeded on 96-well plates (V-shape) and washed twice with the same buffer. Staining was performed by resuspending cells in different combinations of antibodies or as single-color stainings for compensation. Cells were incubated at 4°C for 30 min and washed twice with staining buffer. Monoclonal antibodies (mAbs): CD3-SPRD, CD4-PE, CD8a-FITC, and Bu-PE were purchased from Southern Biotech (Birmingham, AL). All the antibodies were titrated to determine the optimal staining concentration of each one. Positive cells were analyzed with a FACSCalibur flow cytometer (BD Biosciences, San Jose, CA) and CellQuest software. Lymphocyte gates were defined by the forward/side scatter characteristics of the cells, and 30,000 events were analyzed for each sample.

### 2.10. RNA Isolation and cDNA Synthesis

Total RNA from bursal tissue was isolated using TransZol solution (TransGen Biotech, Beijing, China) according to the manufacturer's guidelines. RNA concentration and purity were measured using a Nanodrop N100 (Thermo Scientific^TM^, Wilmington, USA) and agarose gel electrophoresis. One *μ*g of RNA sample was reverse transcribed into cDNA using the MMLV enzyme (Promega, USA) in a 20 *μ*L reaction mixture. A cDNA synthesis reaction was performed in a thermal cycler (Biometra, USA) according to the manufacturer's guidelines.

### 2.11. Humoral Immune Response

Sera obtained from chickens were tested for specific anti-IBDV/VP2 antibodies using an indirect ELISA based on IBDV subviral particles (SVP) developed in our laboratory [35]. Briefly, 96-well Maxisorp™ Nunc™ flat-bottom plates (Thermo Scientific, USA) were coated with 95 ng of SVP per well in 0.1 M carbonate-bicarbonate buffer, pH 9.6, overnight at 4°C. After blocking with 5% skim milk in PBS-T-ENS (0.05% Tween 20, 5% equine normal serum), plates were subsequently incubated with a 1 : 400 dilution of sample sera, washed and incubated again with a 1 : 4000 dilution of goat antichicken IgG antibodies coupled to horseradish peroxidase (Bethyl Laboratories, USA). Revealing step was performed using ABTS substrate (Sigma–Aldrich, USA)-H_2_O_2_ in citric acid buffer, pH 5. Reading was done at 405 nm after 20 min of incubation. Samples with absorbance above the cutoff value 0.249 were considered positive. Results were expressed as percentage of positivity (PP) using the following formula:

(2) $$\begin{array}{c} \text{PP}=\left[\frac{\left(A_{405\text{nm}}S-A_{405\text{nm}}\text{NC}\right)}{\left(A_{405\text{nm}}\text{PC}-A_{405\text{nm}}\text{NC}\right)}\right]\times 100\text{,} \end{array}$$

where PC is the positive control, NC is the negative control, and S is the sample.

Specific anti-NDV IgG was measured with an in-house ELISA. Briefly, 96-well Maxisorp™ Nunc™ flat-bottom plates (Thermo Scientific, USA) were coated with purified La Sota NDV [36] in 0.1 M bicarbonate buffer pH 9.6 overnight at 4°C. After blocking with 4% skim milk in PBS-T-SNE (0.05% Tween 20, 0.5% equine normal serum), they were subsequently incubated with the samples, and with goat anti-chicken IgG antibodies coupled to horseradish peroxidase (Bethyl Laboratories, Inc.). ABTS 2,2′-azino-bis (3-ethylbenthiozoline-6-sulfonic acid), diammonium salt-H_2_O_2_ in citric acid buffer pH 5 were added to each well as substrate. The absorbance was measured at 405 nm, and results were expressed as percentage of positivity (PP) using the following formula:

(3) $$\begin{array}{c} \text{PP}=\left[\frac{\left(A_{405\text{nm}}S-A_{405\text{nm}}\text{NC}\right)}{\left(A_{405\text{nm}}\text{PC}-A_{405\text{nm}}\text{NC}\right)}\right]\times 100\text{,} \end{array}$$

where PC is the positive control, NC is the negative control, and S is the sample.

### 2.12. Seroneutralization Assay

The seroneutralization assay was performed as previously described [37]. Briefly, sera were inactivated for 30 min at 56°C, two-fold serially diluted in culture medium (50% MEM-D, 50% MEM-E, Hepes 1X, pH 7.4), and incubated with 100 TCID_50_ of IBDV strain Winterfield for 1 h at 37°C in 96-well plates. Subsequently, 100 *μ*l of a cell suspension of 1 × 10^6^ VERO cells/ml was added to each well. Cells were cultured at 37°C, with 5% CO_2_ for 4 days, when a cytopathic effect was observed. Virus neutralizing antibody titers were calculated as the inverse of the last dilution, showing no cytopathic effect. Two sera belonging to hyperimmunized hens were used as positive controls.

### 2.13. Viral Load

cDNA synthesis and qPCR were performed in a single step reaction utilizing the Luna® Universal Probe OneStep RT-qPCR Kit (New England Biolabs, Massachusetts, USA) according to the manufacturer's protocol. The primers used for retrotranscription and amplification were VP1f: 5′CCAACACACCTCATGATCTC3′ and VP1r: 5′GTCAATTGAGTACCACGTGTT3′, which amplify a product of 222 bp belonging to the VP1 gene of IBDV. The number of viral copies per microgram of RNA was calculated by extrapolation with a standard curve generated by qPCR from ten-fold serial dilutions of a plasmid containing the amplified VP1 fragment, ranging from 10^2^ to 10^9^ copies.

### 2.14. Statistical Analysis

Statistical analyses were performed using one-way ANOVA and mean differences were analyzed with the Tukey test. The Shapiro–Wilk and Levene tests were applied to verify the assumptions. In the cases where heteroscedasticity was detected, the variance structure was modeled. Transformation of data was also performed when normality was not assumed. When assumptions were not fulfilled, the Kruskal–Wallis nonparametric test was applied followed by the Wilcoxon pairwise comparison. All the analyses were done using R 3.4.1 (R core team) and the agricolae package [38].

## 3. Results

### 3.1. Bursa to Body Weight Ratio

An experimental infection of Negra INTA and WL chickens was carried out to evaluate the pathogenesis of an Argentinian field isolate belonging to genogroup 1 and to describe the differences between the chickens' lines.

**Figure 1:**
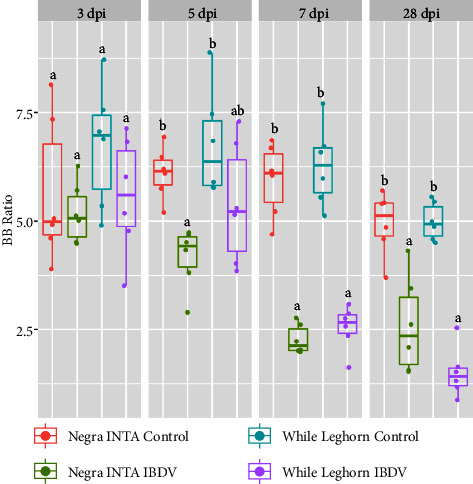
Bursa/body weight (BB) ratios of Negra INTA and White Leghorn chickens infected with IBDV. Chickens were sacrificed and weighted at different times postinfection. Bursae were extracted and also weighted. Individual BB ratios were determined by the formula (bursa weight (g)/body weight (g)) × 1000. The box plots represent data distribution. Different letters indicate significant differences among groups (one-way ANOVA test and Tukey post-hoc test, *p* < 0.05).

IBDV-infected and noninfected chickens were sacrificed at different time points, and their body and bursa weights were measured to obtain the BB ratios which are shown in Figure 1. The bursae of all IBDV treated birds were affected regardless of their breed, given that the BB ratio was lower in the infected birds. No significant differences between the infected groups were detected. While at 5 dpi, the Negra INTA (NI) IBDV group differed significantly from control birds (*p* ≤ 0.05), and no significant differences between treated and control WL chickens were observed until 7 dpi, the time point at which maximum variation was reached in both lines. At day 28 pi, the bursae of infected chickens still showed a reduced BB ratio, and the infected NI group showed a greater dispersion in the BB index compared to the chickens of the WL group, which remained extremely low.

### 3.2. Histopathological Observation of Bursa

**Figure 2:**
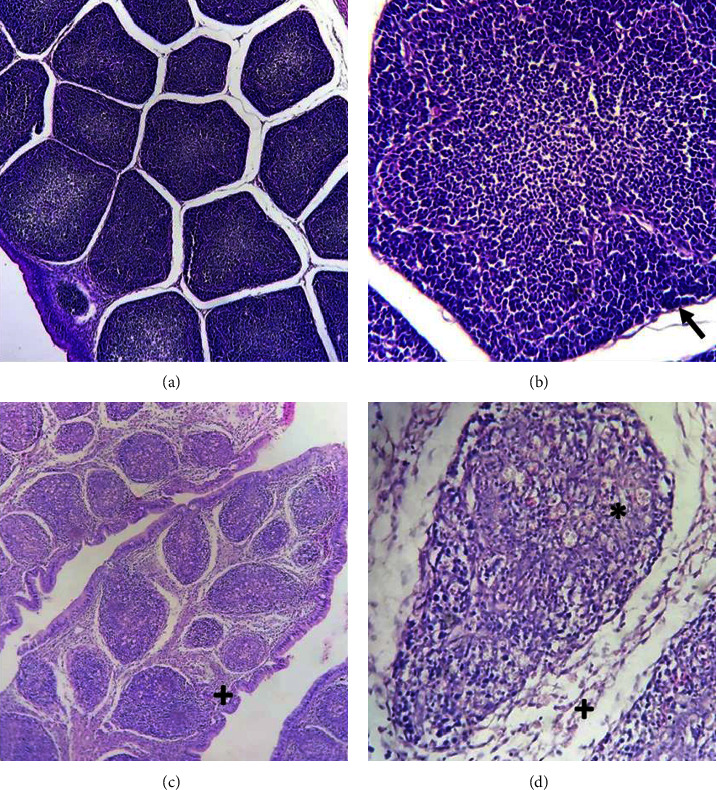
Representative photos of bursal lesions. The arrow indicates the presence of lymphocytes in the cortex, (*∗*) points the absence of lymphocytes in the cortex and (+) shows the inflammatory infiltrate. (a and b) Photographs of a healthy bursa. (c and d) A bursa with the highest score in the measured parameters.

**Figure 3:**
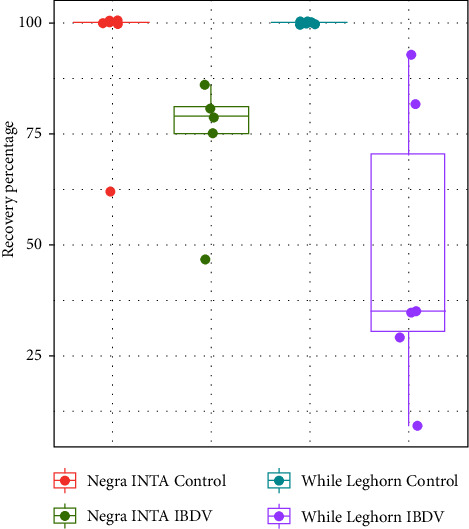
Recovery evaluation of bursal follicles of infected chickens. Bursae collected at 28 dpi were fixed and PAS stained. The number of follicles with and without PAS-positive membranous structures was counted in each sample. Recovery index (RI) was calculated using the following formula: RI = total number of PAS-negative follicles/total number of PAS-positive follicles. Results are presented as the mean of the RI of each group.

At different times postinfection, bursae of treated and nontreated chickens were examined for several lesions as described in the material and methods section.  Table 1 shows the average of scores corresponding to the inflammatory infiltrate and lymphoid depletion of infected chickens from both lines at different dpi. All control birds showed a score 1 (data not shown). In addition, Figure 2 shows representative photos of control and infected bursae where lymphoid depletion and inflammatory infiltrate can be observed. No differences between groups were observed through the time course of the experiment with the exception of Negra INTA's inflammatory infiltrate on day 5, which was significantly higher than the White Leghorn's. Finally, Figure 3 shows the proportion of follicles recovered at 28 dpi. There were no significant differences between groups; however, the infected White Leghorn group presented a greater dispersion of the results, suggesting a tendency toward weaker recovery ability.

### 3.3. Chicken Humoral Immune Response

**Figure 4:**
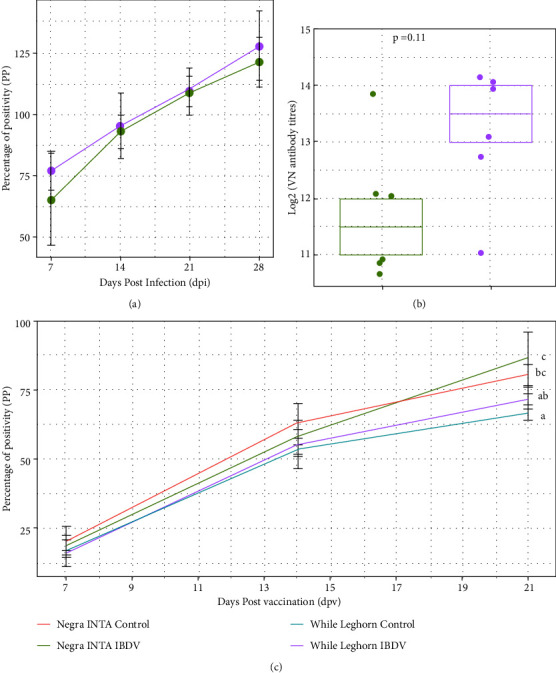
Anti-IBDV and anti-NDV humoral immune responses. (a) Percentage of positivity (PP) of anti-IBDV antibodies at 7, 14, 21, and 28 dpi obtained with an indirect ELISA. The mean PP values ± SD are shown for each group at the different time points; data from the negative controls of infection were less than 15% (data not shown). (b) IBDV neutralizing antibody titers in IBDV-infected chickens at 28 dpi, calculated as the inverse of the last dilution showing no cytopathic effect. Log2 of the median of each group is shown and the comparisons were made using Wilcoxon–Mann–Whitney, *p* < 0.05. (c) Percentage of positivity (PP) of anti-NDV antibodies in sera collected at 7, 14, and 21 days postvaccination with NDV measured with an in-house ELISA. Data from the negative controls of NDV vaccination were less than 15% (data not shown). Different letters indicate significant differences among groups at day 21 (one-way ANOVA test and Tukey post-hoc test, *p* < 0.05).

In order to describe the immunocompetence of the lines after IBDV infection, serum samples were weekly collected at 7, 14, 21, and 28 dpi and assayed to detect anti-IBDV and anti-NDV specific IgG antibodies. In addition, sera obtained at 28 dpi were evaluated by a viral neutralization (VN) assay. Figure 3 shows that the level and kinetics of the anti-IBDV antibodies response were similar between White Leghorn (WL) and Negra INTA (NI) chickens during the time course of the experiment (Figure 4(a)). Also, sera from both lines of chickens showed viral neutralizing activity; although no significant differences were detected, WL chickens reached higher neutralizing titers (Figure 4(b)). Regarding the humoral response against NDV, chosen as a nonrelated antigen, control and infected NI chickens showed significant differences with the WL chickens at 21 dpv (Figure 4(c)). Infected NI birds reached the highest titers against the unrelated antigen (NDV), meanwhile nonvaccinated chickens did not show antibodies against NDV (PP < 15%).

### 3.4. Viral Load

**Figure 5:**
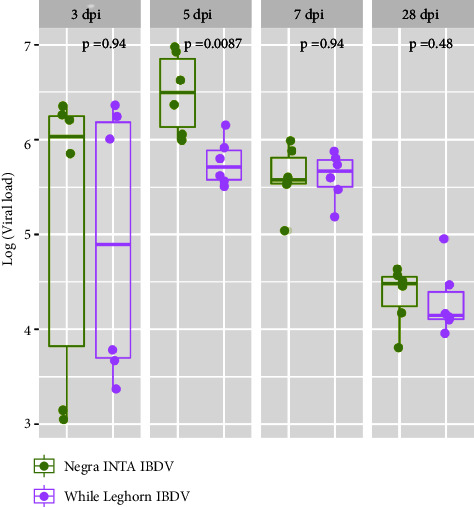
Viral load in the bursa of IBDV-infected chickens. Bursae were collected at 3, 5, 7, and 28 dpi and total RNA was extracted. The number of IBDV viral copies/*μ*g of bursal RNA was estimated by RT-qPCR. The log of individual values (dots) as well as box plots representing data distribution are shown for each group. The *p* value is shown for each day (*t*-test).

In order to compare the ability of IBDV to reach and/or replicate in the bursa of both lines, the viral load was quantified by RT-qPCR at 3, 5, 7, and 28 dpi using total RNA from bursal samples (Figure 5). The viral genome was detected in all the samples except the negative controls (data not shown). At 3 dpi, great variability was observed within each of the infected groups, and no significant differences were found between them. At 5 dpi, the maximum number of viral genomes was observed in the NI group, presenting significant differences in comparison with the WL group (*p* < 0.05). On day 7 pi, both groups showed very similar viral load without finding significant differences. Finally, on day 28, both groups showed an important decrease in viral load values without significant differences between both lines.

### 3.5. Flow Cytometry

**Figure 6:**
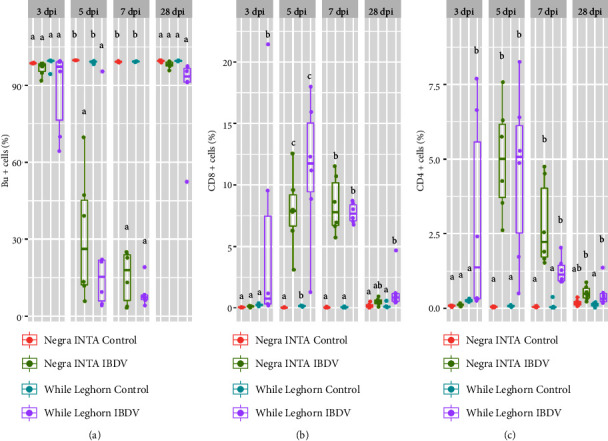
Analysis of lymphocyte populations by flow cytometry. Mononuclear cells were isolated from bursae, stained with different combinations of antibodies and analyzed by flow cytometry. The lymphocyte population was gated according to their size and complexity. The results show the percentage of cells in each of the groups analyzed. (a) Percentage of Bu+, (b) CD8+, and (c) CD4+ cells in the bursa. Percentages values (dots) as well as box plots representing data distribution are shown for each group. Different letters indicate significant differences among groups (one-way ANOVA test and Tukey post-hoc test, *p* < 0.05).

After IBDV infection, a great depletion of Bu+ IgM+ lymphocytes takes place in the bursa [39]. In addition, infiltration of both CD3+ lymphocytes, particularly CD8+ cytotoxic T cells, occurs to perform viral clearance. It is also known that excessive infiltration can cause damage to the bursa [40, 41]. At 3, 5, 7, and 28 days postinfection, bursal lymphocytes were isolated and stained to be studied by flow cytometry. Our results showed significant differences between the control groups and infected chickens from each of the analyzed lines (Figure 6). In both lines, the decrease in Bu+ cells was seen as soon as 3 dpi, with a maximum at 7 dpi and a total recovery by 28 dpi compared to the unchallenged birds (Figure 6(a)). At 3 dpi, the WL infected group showed a higher proportion of CD8+ and CD4+ cells (Figures 6(b) and 6(c)). With respect to the other time points, the chickens did not show great differences. On the other hand, we could see that control WL and NI chickens significantly differed in the proportion of CD3+ CD8+ cells at 5 dpi. At 28 dpi, NI-infected chickens did not show differences compared to their control group; meanwhile, WL infected birds still had a higher percentage of CD8+ and CD4+ lymphocytes.

## 4. Discussion

Since 1997, in Argentina, the PROHUERTA program has overseen distribution of the Negra INTA hybrid chicken with the purpose of producing backyard eggs for self-consumption. These autosexative hens were developed at the INTA Pergamino experimental station, where the genetic core for their production is maintained. There are well documented records of the productive parameters of this hybrid [29], but, until now, no evaluation on disease resistance features was performed. The objective of our work was to characterize and compare the pathogenesis and the immune response induced by an Argentinian IBDV isolate in Negra INTA and White Leghorn lines after infection.

It is known that IBDV is one of the many viruses that cause immunosuppression since it has specific tropism for B cells. It is also known that classic field strains (genogroup 1) cause severe atrophy of the bursa, which is the specific site of production and maturation of B cells. In this study, the immune response of Negra INTA chickens to a local Argentine isolate of IBDV belonging to genogroup 1 was analyzed for the first time and compared to White Leghorn chickens' responses.

In this work, we used 28-day-old chickens to mimic a natural IBDV infection since it is known that the susceptibility period begins at 21 days after hatch when maternal antibodies decrease [42, 43]. To compare features of the immune response to the infection in different animal lines, chicks belonging to a commercial line of the White Leghorn breed and chicks of the Negra INTA (NI) line were used. The assay consisted of infecting chickens with a single dose of IBDV on day 28 of life and sacrificing them at 4 time points (3, 5, 7, and 28 dpi) to evaluate several parameters. In previous studies, we challenged chickens with 10^4^ ELD_50_ of the classic strain. Such a dose had caused disease in 14-day-old chicks in the presence of maternal antibodies [44]. However, in the present study, we chose a dose of 10^3^ ELD_50_ since the birds lacked specific defenses against IBDV. Both groups of chickens were found to be susceptible to IBDV infection as expected, showing similar results in the body-to-bursa ratio at 3, 7, and 28 dpi. On day 5 pi, the infection impact on the body-to-bursa ratio was greater in NI chickens compared to WL chickens. These differences disappeared at 7 dpi since both groups differed from their corresponding controls but not between them.

It has been suggested that the degree of lesions and the amount of virus in the bursa may differ between breeds [24, 45, 46]. Dobner suggested that the presence of a higher viral load in the bursa corresponds to more severe microscopic lesions [26]. In our study, we were able to corroborate this last statement since the highest viral load was measured in the NI group at 5 dpi and that was the time when the most severe histological lesions were found in this group. Furthermore, this was the only time when we detected differences between chicken lines. Thus, there were no differences between the viral load and the lesions on the other days analyzed. There is evidence that at early times of the infection, presence of viral genome is observed in susceptible lines, but it cannot be detected in resistant lines [24]. In our study, all the infected chickens showed viral load in their bursae at every analyzed time.

Regarding the specific serology against IBDV, no significant differences were observed between the groups. Since it is well known that neutralizing antibodies are responsible for providing humoral immunity against IBDV [47, 48], we evaluated the neutralizing activity of anti-IBDV antibodies in both lines. The results showed that, although both groups presented high titers of neutralizing antibodies, the White Leghorn chickens showed a tendency to develop higher neutralizing titers. This result could be related to the ability to develop a more efficient immune response to IBDV infections after vaccination.

It has been reported that the older the chickens, the lower the degree of humoral immunosuppression caused by IBDV infections [49, 50]. We infected 28-day-old chickens and vaccinated them with an inactivated Newcastle vaccine at 7 dpi. We could not detect significant differences in the humoral immune response between infected and uninfected birds, most likely due to their advanced age. Surprisingly, we observed that chickens belonging to the Negra INTA hybrid presented a higher titer of specific antibodies against the Newcastle virus at 21 dpv, regardless of IBDV treatment. This feature may represent an advantage for the Negra INTA breed since it reported a positive correlation between the antibody levels and the survival of hens to the laying cycles [51]. Negra INTA could be more suitable to resist the conditions of industry-specific overcrowding; however, further investigations are needed in this area.

In our study, all IBDV-infected birds showed a marked reduction in Bu+ B cells, in addition to the typical lesions of the disease so well characterized [52]. It was reported that the Malaysian breed presented the lowest percentage of IgM+ B-lymphocytes at 1 and 3 days post-IBDV infection. Here, we show that B cell reduction was maximum at 5 and 7 dpi and that a total recovery was observed at 28 dpi. However, this parameter did not correlate with a decrease in viral load or recovery of histopathological damage to the bursa [53].

It has been demonstrated that T cell infiltration is necessary to stop IBDV infection, although excessive infiltration is capable of exacerbating bursal lesions. Moreover, the induction of local inflammation can delay the recovery of the bursa [40, 41]. We observed that infected chickens had similar degrees of CD4+ and CD8+ cells infiltration. In agreement with Farhanah et al. [53], we found that the maximum percentage of CD4+ cells in bursa was observed at 5 dpi, but the percentage was lower compared to that study, which could be attributed to the challenge strain or the viral dose. The percentage of CD8+ cells over time followed a similar pattern to that of CD4+, reaching a maximum at 5 dpi. Regarding control groups, NI chickens showed a lower proportion of CD8+ cells at baseline, which could indicate a greater degree of susceptibility to IBDV infection.

Regarding bursal recovery, there are few studies that evaluate this parameter beyond the period of susceptibility [26]. We observed that CD4+ and CD8+ cell infiltration was significant even at 28 dpi in the WL group compared to their uninfected control.

In conclusion, the infection with the local isolate of IBDV belonging to the genogroup 1 produced similar effects in both Negra INTA and White Leghorn chickens, with some punctual differences, especially at 5 dpi. On the other hand, our study revealed that the lines differ in their basal proportions of lymphoid cells in the bursa. However, these differences might not be substantial enough to produce a differential response against the infection.

This study gives us a guideline that IBDV infection assays carried out on White Leghorn chickens around the world are comparable with the Negra INTA chickens that are widely used in poultry production in Argentina.

## 5. Conclusions

The infection with a local isolate of IBDV belonging to the genogroup 1 produced similar effects in both Negra INTA and White Leghorn chickens. All chickens responded similarly regarding IBDV pathogenesis and immune response.

## Figures and Tables

**Table 1:** Histopathological bursal lesions of infected chickens.

Lines		Dpi			
		3	5	7	28
White Leghorn	Lymphoid depletion	2.67 ± 1.86a	4.17 ± 0.98a	4.83 ± 0.41a	3.33 ± 0.52a
Negra INTA	Lymphoid depletion	2 ± 1.67a	4.8 ± 0.45a	5 ± 0a	4 ± 1.67a
White Leghorn	Inflammatory infiltrate	2.5 ± 1.76a	3.5 ± 1.38a	3.83 ± 0.75a	3.17 ± 0.75a
Negra INTA	Inflammatory infiltrate	2.17 ± 1.83a	5 ± 0b	3.83 ± 0.75a	2.67 ± 1.03a

Bursae were extracted at 3, 5, 7, and 28 dpi, fixed, and stained for histological evaluation. Lymphoid depletion an inflammatory infiltrate were evaluated and scored for White Leghorn and Negra INTA chickens. Results are presented as the mean ± SD of the lesion scores registered for each group. a, b indicate significant differences between genotype groups per day (Wilcoxon–Mann–Whitney, *p* < 0.05).

## Data Availability

The data used to support the findings of this study are available from the corresponding authors upon request.
